# Involvement of People With Dementia in the Development of Technology-Based Interventions: Narrative Synthesis Review and Best Practice Guidelines

**DOI:** 10.2196/17531

**Published:** 2020-12-03

**Authors:** Harleen Kaur Rai, Aline Cavalcanti Barroso, Lauren Yates, Justine Schneider, Martin Orrell

**Affiliations:** 1 Division of Psychiatry and Applied Psychology Institute of Mental Health University of Nottingham Nottingham United Kingdom; 2 School of Sociology and Social Policy Law and Social Sciences Building University of Nottingham Nottingham United Kingdom; 3 Department of Clinical, Education and Health Psychology University College London London United Kingdom

**Keywords:** dementia, technology, co-production, participation, development

## Abstract

**Background:**

Technology can be helpful in supporting people with dementia in their daily lives. However, people with dementia are often not fully involved in the development process of new technology. This lack of involvement of people with dementia in developing technology-based interventions can lead to the implementation of faulty and less suitable technology.

**Objective:**

This systematic review aims to evaluate current approaches and create best practice guidelines for involving people with dementia in developing technology-based interventions.

**Methods:**

A systematic search was conducted in January 2019 in the following databases: EMBASE (Excerpta Medica database), PsycINFO, MEDLINE (Medical Literature Analysis and Retrieval System Online), CINAHL (Cumulated Index to Nursing and Allied Health Literature), and Web of Science. The search strategy included search terms in 3 categories: *dementia*, *technology*, and *involvement in development*. Narrative synthesis wove the evidence together in a structured approach.

**Results:**

A total of 21 studies met the inclusion criteria. Most studies involved people with dementia in a single phase, such as development (n=10), feasibility and piloting (n=7), or evaluation (n=1). Only 3 studies described involvement in multiple phases. Frequently used methods for assessing involvement included focus groups, interviews, observations, and user tests.

**Conclusions:**

Most studies concluded that it was both necessary and feasible to involve people with dementia, which can be optimized by having the right prerequisites in place, ensuring that technology meets standards of reliability and stability, and providing a positive research experience for participants. Best practice guidelines for the involvement of people with dementia in developing technology-based interventions are described.

## Introduction

### Background

Technology can be used to address some of the challenges of dementia care and enable people with dementia to maintain their independence for as long as possible [1]. Despite the wide variety of technology available (eg, reminder devices, touchscreen devices and apps, and computerized cognitive and physical interventions) [2], there is a lack of evidence on their efficacy, and many interventions are either in the development phase or in a prototype phase [3]. Moreover, there has been little involvement of people with dementia in the development of technology-based interventions [4]. Possible reasons for this lack of involvement include stigma, concerns about the frailty of older people, and the anticipated distress among participants caused by trying out less developed information technology [5]. Underdeveloped technology-based interventions with inadequate involvement could have residual faults and could potentially make early prototypes harder to operate for people with dementia and lead to people with dementia being reluctant to use them [5]. Consequently, technologies are being developed that are neither user-friendly nor fit for the purpose of supporting people with dementia [3,4]. Technology that is faulty or poorly designed may not be useful in supporting people with dementia.

A previous systematic review showed that people with dementia are able to provide useful feedback, such as comments on screen size, language difficulties, and the importance of personalization on private spaces of websites, which help to improve the quality of the intervention [4]. This approach improves the usability and acceptability of the technology-based interventions [4] and generates enjoyment and enthusiasm in participants with dementia [6,7]. However, Span et al [4] only reviewed papers up to 2010, and subsequently, many innovations in technology have taken place. Furthermore, Astell et al [8] and Span et al [4] assert that to optimize technology by ensuring the needs and preferences of people with dementia are addressed, it is crucial to implement a participatory process in which people with dementia are involved throughout the development process [4,8].

Information on how to optimize the involvement of people with dementia is dispersed, and there is a clear need to bring the evidence together in a systematic manner through an appraisal of the involvement of people with dementia in the development of technology-based interventions and guidelines on how to best facilitate and optimize this involvement.

### Objectives

This narrative synthesis systematic review sets out to appraise the methods used by applying existing frameworks, such as the Medical Research Council (MRC) framework for the evaluation of complex interventions and the Centre for eHealth Research (CeHRes) roadmap [9,10], and to create best practice guidelines on how to better involve people with dementia in developing technology-based interventions accompanied by a logic model.

## Methods

### Narrative Synthesis

Narrative synthesis is “an approach to the systematic review and synthesis of findings from multiple studies that relies primarily on the use of words and text to summarize and explain the findings” [11]. Narrative synthesis can be used to address a multitude of questions regarding the effectiveness of interventions, including what works but also why and how. Narrative synthesis is preferred for this review as it can be used to convert the evidence into clear and structured best practice guidelines on how to facilitate the participation of people with dementia in the development of technology-based interventions. The approach consists of 4 elements: theory development, developing a preliminary synthesis, exploring relationships within and between studies, and assessing the robustness of the synthesis.

#### Element 1: Theory Development

Theory development underpins the systematic review by supporting the development of the review question and the types of studies to be included. Our starting point is the desirability of end user involvement in technology development. Several studies suggest that feedback from people with dementia can lead to improvements in the overall quality of the technology [4,8]. This would result in more useful and suitable pieces of technology and would also increase the willingness to use the technology. Furthermore, the involvement of end users in developing technology could also support the implementation of a technology in the future, leading to a better range of technology to improve the quality of life of people with dementia. Therefore, we only include studies that clearly illustrate how feedback was gathered from people with dementia during development. This would exclude studies with a sole focus of including participants as objects of studies where no meaningful involvement has taken place. The narrative synthesis undertaken here will contribute to the refinement of our theoretical starting point and support the application of the findings of this review [11].

#### Element 2: Developing a Preliminary Synthesis

The preliminary synthesis develops an initial description of the results of the included studies, organized in a manner such that a pattern can be described in terms of effects or impact [11]. This can be done through the use of textual descriptions, grouping and clusters, and tabulation. This preliminary synthesis is necessary to inform the next steps of the narrative synthesis.

#### Element 3: Exploring Relationships Within and Between Studies

The patterns that emerge from the preliminary synthesis are subjected to a more detailed analysis in which the reviewers move toward exploring the relationships within and across the included studies [11]. The relationships between the characteristics and reported findings of different studies are reviewed. This element of narrative synthesis will help identify the factors that may have influenced the results and will seek to provide an explanation of how and why a particular intervention works [11]. The methods used here include qualitative case descriptions and the development of a conceptual model based on the grouping of study findings. This will help to structure the inferences drawn from our results.

#### Element 4: Assessing the Robustness of the Synthesis

The final element of narrative synthesis sets out to review the trustworthiness of the results [11]. The trustworthiness of the synthesis is affected by the quality and quantity of the evidence on which the synthesis is built and by the methods used. Therefore, an appraisal is undertaken to judge the strength of the evidence for the findings and to generalize them to different populations and contexts [11].

### Electronic Searches and Screening

This review was registered in the International Prospective Register of Systematic Reviews (PROSPERO) under protocol number CRD42017068933. After conducting 2 pilot searches, we systematically searched the following databases: EMBASE (Excerpta Medica database), PsycINFO, MEDLINE (Medical Literature Analysis and Retrieval System Online), CINAHL (Cumulated Index to Nursing and Allied Health Literature), and Web of Science in January 2019. Studies published between 2000 and 2019 were considered. The search strategy consisted of combinations and variations of search terms in the following 3 key categories: *dementia, technology*, and *involvement in development*. Involvement terms also included *codesign, participatory research,* and *user participatory development.*

After removal of the duplicates, a 3-stage screening process was independently conducted by 2 review team members (HR and AB): (1) titles were screened for relevance to the review question, and irrelevant studies were archived; (2) abstracts were assessed (referring to the full text whenever necessary to clarify the relevance of the study); and (3) quality assessment of the remaining studies was conducted (see the *Data Extraction and Study Quality Assessment* section). The reasons for exclusion were recorded by archiving the excluded studies in relevant folders in EndNote (Clarivate Analytics). In case of disagreement between the 2 reviewers, a third review team member was consulted (LY). Additional studies from the review by Span et al [4] were distributed separately among 4 review members (AB, JS, HR, and LY) for data extraction and quality assessment. The reference lists of studies that passed the quality assessment were reviewed to ensure the inclusion of other relevant papers.

### Criteria for Inclusion and Exclusion of Studies

The inclusion and exclusion criteria of the studies were as follows:

- Types of participants: people with a diagnosis of dementia, irrespective of age, type of dementia, or stage of the disease.
- Types of intervention: involvement of people with dementia in the development process of a technology-based intervention.
- Types of studies: quantitative, qualitative, and mixed methods studies published from the year 2000 onward as an English language journal paper with sufficient study quality (a minimum of 5 criteria met as assessed with the Critical Appraisal Skills Programme [CASP] guidelines or 50% of the criteria met as assessed with the Downs and Black checklist).

### Description of Development Phases

The development process of a technology-based intervention consists of several stages. To identify the key stages of technology development for this review, we employed the MRC framework together with the CeHRes roadmap [9,10]. Both frameworks focus on developing interventions; however, although the MRC framework is more widely used for developing complex interventions, the CeHRes roadmap has a focus on digital health interventions (Table 1).

**Table 1:** Description of the Medical Research Council framework and the Centre for eHealth Research roadmap.

Phase	Medical Research Council framework	Centre for eHealth Research roadmap
Development	Single phase Identifying evidence base (eg, systematic review) Identifying or developing theory (eg, scope existing theories and interviewing stakeholders) Modeling process and outcomes (eg, undertaking a pretrial economic evaluation, focus groups, surveys, and case studies)	Multiple phases such as contextual inquiry, value specification, and design Identifying problems and needs of intended users (eg, literature review, field observations, interviews, and workshops) Determining the most favorable solutions based on the values of the stakeholders Building prototypes to fit values and user requirements (eg, focus groups and field testing)
Feasibility and piloting	Specific phase for feasibility and piloting Activities consist of testing procedures for acceptability, determining appropriate sample size, and estimating rates of recruitment	N/A^a^ (can be part of the design phase)
Evaluation	Assessing clinical and cost-effectiveness (eg, randomized controlled trial) Understanding processes (process evaluation)	Summative evaluation Assessment of the impact of eHealth technologies in clinical, organizational, and behavioral terms
Implementation	Getting evidence into practice Surveillance, monitoring, and long-term outcomes	Operationalization Activities to introduce, adopt, and employ the technology in practice (eg, creating a business model)

**^a^**N/A: not applicable.

### Data Extraction and Study Quality Assessment

A standardized data extraction form was developed by the primary researcher (HR), in which the review team members recorded the extracted data from the final studies, including the study quality rating (Multimedia Appendix 1) [6,7,12-30].

Quality was assessed using the CASP guidelines. These guidelines consist of 8 checklists for various types of studies and include items that assess multiple aspects of research (eg, recruitment, risk of bias, confounders, data collection, data analysis, results, and implications) [31]. The studies were rated as high quality if 8 or more criteria were met, medium quality if 5 to 7 criteria were met, and low quality if 4 or less criteria were met [32]. Studies that did not meet the criteria for assessment with the CASP guidelines were assessed with the Downs and Black checklist [33]. This checklist is appropriate for both randomized and nonrandomized studies and consists of 27 items over 5 domains (reporting, external validity, internal validity—bias, internal validity—confounding, and power). The maximum score was dependent on the study design; however, each study was rated as high quality if it met over 81% of the criteria, medium quality if it met over 66% to 80% of the criteria, fair quality if it met over 51% to 65% of the criteria, and low quality if it met 50% of the criteria or less [34]. Studies considered to have low quality were excluded. The review team members independently assessed the studies for sufficient study quality. Any differences in judgment between the 2 reviewers were resolved by a third review team member.

### Consultation With the Patient and Public Involvement Group

One reviewer (HR) presented the findings at a patient and public involvement (PPI) consultation meeting on 2 different occasions. This PPI group is run on a monthly basis at the Institute of Mental Health in Nottingham. The aim of both meetings was to gain insights into people’s own views on optimal involvement in developing technology-based interventions; their feedback and comments on the findings; and, more specifically, their feedback on the guidelines drafted by the authors. This feedback would then be integrated within the findings of this review and used to strengthen the best practice guidelines.

The first meeting was attended by 2 people with dementia, 1 carer, 1 volunteer, and 1 researcher and lasted for 45 min. The second meeting was attended by 2 people with dementia, 2 carers, 1 volunteer, and 4 researchers and lasted for 25 min. After a brief introduction to the review and its findings, the best practice guidelines were presented one at a time on a projector. In the first meeting, printed handouts were distributed to each participant. A short discussion in terms of relevance and accuracy encompassed each guideline, and notes were taken throughout the meeting.

## Results

The *Results* section comprises the second element of narrative synthesis: developing a preliminary synthesis.

### Search Results

**Figure 1:**
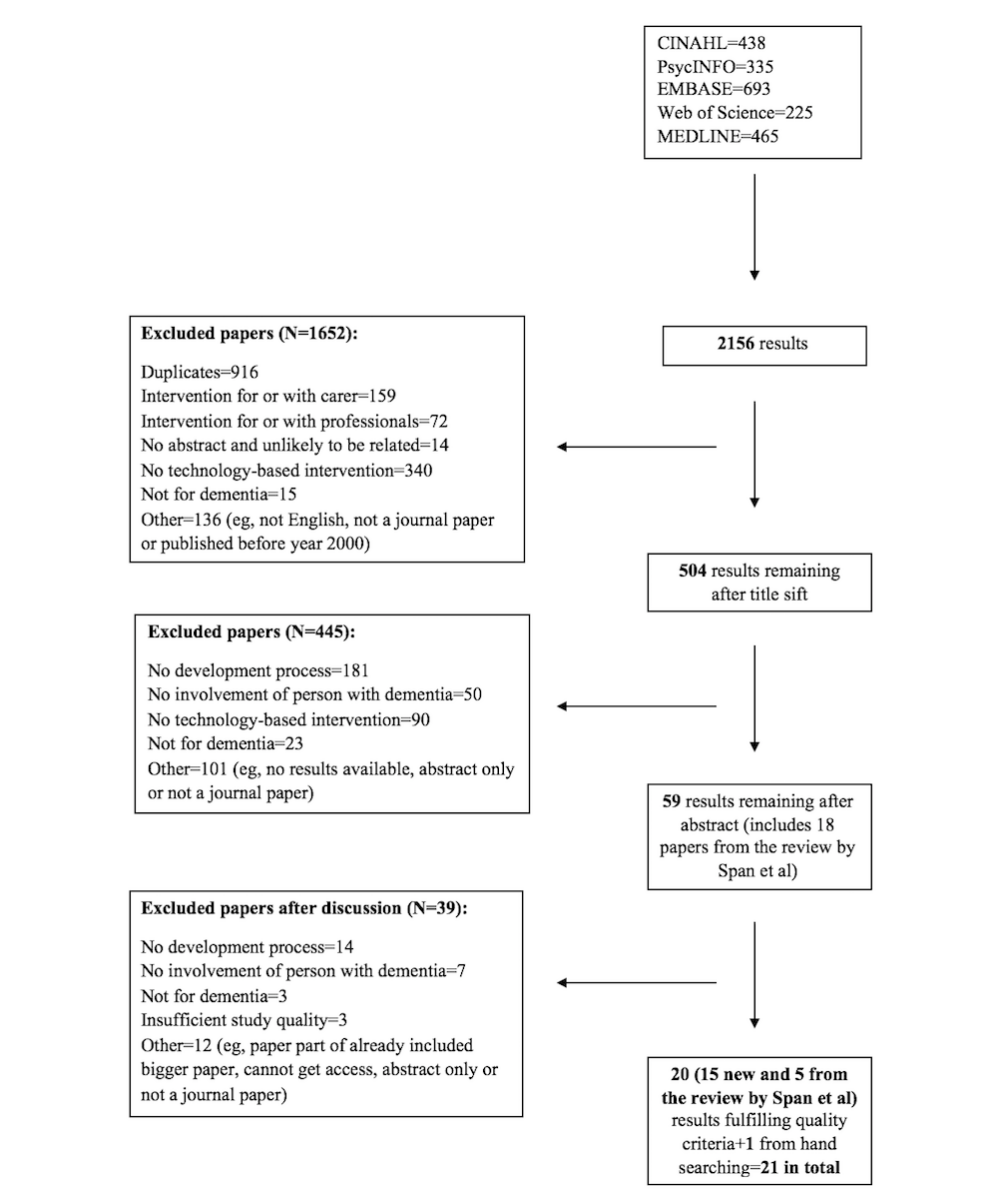
Flowchart of study selection. CINAHL: Cumulated Index to Nursing and Allied Health Literature; EMBASE: Excerpta Medica database; MEDLINE: Medical Literature Analysis and Retrieval System Online.

A total of 2156 potentially relevant titles were identified across the 5 databases (Figure 1). Removal of duplicates and screening of titles, abstracts, and full texts resulted in 20 studies that met the inclusion criteria. The most frequent reasons for exclusion were the lack of a technology-based intervention and absence of a development process. Additional hand searching led to the inclusion of one other study, making up a total of 21 studies. This study came from a review by Span et al [4], which was not captured by the current search strategy. Other studies from the same review not captured by the search strategy (n=7) were excluded because they did not meet the inclusion criteria (eg, not a journal paper or low study quality). The reference lists of studies passing the quality assessment were reviewed to ensure that any other relevant studies would be included.

### Description of Included Studies

The main study characteristics of all 21 studies included study sample and design, description of the technology-based intervention, and rating of study quality (Multimedia Appendices 1 and 2) [6,7,12-30]. Using the CASP Qualitative checklist, 11 studies were assessed as high quality and 8 studies were assessed as medium quality. Only 1 study was assessed with the CASP Randomized Controlled Trial (RCT) checklist, which met 7 of 11 criteria [12]. Another study was assessed using the Downs and Black checklist. It was rated as fair quality, meeting 65% of the criteria for a before and after follow-up study [13]. Most studies were conducted in Europe (n=17), 3 studies took place in Australia [13-15], and 1 was conducted in Canada [16].

A majority of the studies adopted a purely qualitative methodology (n=14). A total of 6 studies employed a mixed methods approach, of which 1 combined qualitative methods with a controlled trial [12]. Only 1 study adopted a purely quantitative methodology [13]. The studies described a variety of technology-based interventions, including communication aids, music tools, devices to support activities of daily living, reminder systems, and tracking devices. In the majority of the studies, people with dementia were involved along with carers or other professionals who either supported the person with dementia in their involvement or provided separate input themselves (n=17). Only 4 studies solely included people with dementia [13,17-19].

### Methods of Involvement and Key Findings

The methods used to involve people with dementia along with the phases of the MRC framework and CeHRes roadmap are summarized in Textbox 1, allowing for an initial synthesis of the findings.

**Methods used to involve people with dementia in the studies (N=21) according to the Medical Research Council framework phases.**

Development (contextual inquiry, value specification, and design)

- Behavioral observations [17,20], focus groups [6,7,18,21-24], interviews [6,18,19,22,24-27], workshops [7,25], questionnaires [17], and user tests [6,7,18-20,22]

Feasibility and piloting

- Behavioral observations [13-16,28,29], interviews [14-16,25,27,29,30], questionnaires [13,16,29,30], field testing [18,25,29], and technical system usage [27]

Evaluation (summative evaluation)

- Randomized controlled trial [12], focus groups [12], interviews [12], and questionnaires [12]

Implementation (operationalization)

- Not applicable

### Development Phase (n=10)

**Figure 2:**
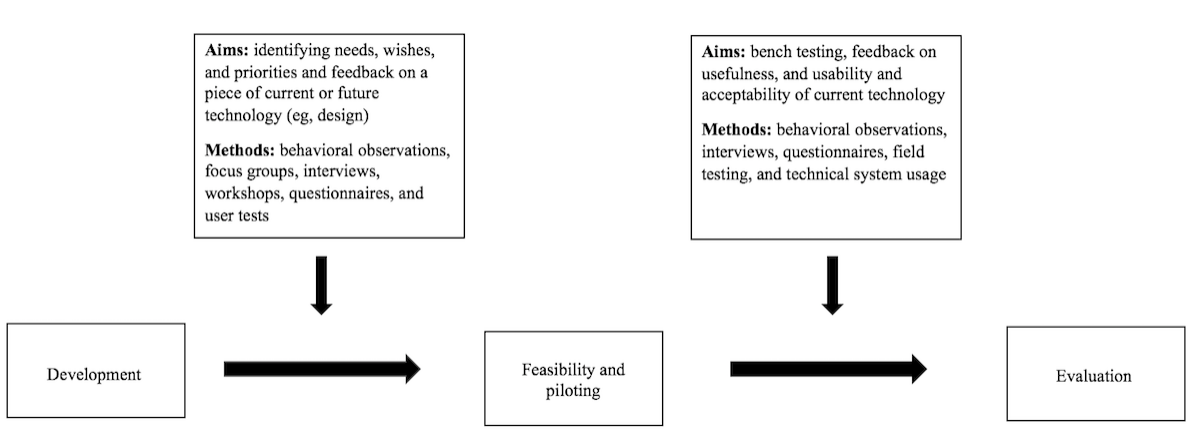
Aims and methods of involvement along the development stages of technology according to the Medical Research Council framework.

A total of 10 studies involved people with dementia solely in the development phase, which coincides with the contextual inquiry, value specification, and design phase of the CeHRes roadmap. The majority of these studies primarily employed qualitative methods, such as focus groups and semistructured interviews. At times, these were accompanied by user tests, observations, and questionnaires. Textbox 1 provides an overview of all methods used in the development phase. The aims of the studies ranged from identifying the needs, wishes, and thoughts of people regarding certain areas for development (eg, independence or cognitive reinforcement) to gaining feedback on the design of future or existing technologies (Figure 2).

#### Needs Assessment and Design of Future Technology

A total of 2 studies included needs assessments, followed by discussions about the design of future technology using qualitative methods. Boman et al [21] used focus groups to capture experiences, expectations, and thoughts concerning a videophone and its design concepts. The design had to be flexible to meet the needs of people with dementia, be easy to use, and not look like assistive technology. Another example is the study by Robinson et al [7] who also used focus groups to elicit views and concerns about independence from people with dementia and carers. A list of priorities was derived from the findings. Areas for functional improvement included 2-way communication, flexibility of functionality, and something to *guide* them home when outside. Workshops were then used to identify the preferred design and functionality aspects of future technologies. Finally, user tests were performed with paper prototypes until 2 fully functional devices were developed.

#### Needs Assessment and User Tests

In 3 other studies, needs assessments were followed by user tests with functional technologies. Orpwood et al [19] used interviews with users (user surveys) to compile a wish list of issues that were of importance in maintaining the quality of life of people with dementia. A large list of potential technologies that could address these issues was generated. The following 4 technologies were selected for initial development: a music player, a device to reduce social isolation, a conversation prompter, and a device to support sequences of tasks. Useful design guidelines were derived from user tests, particularly for intuitive control interfaces (eg, controls need to stand out, be big, and simple).

Touch screens appear to be very intuitive, and prompts seem to be more effective than verbal or visual instructions. Hanson et al [6] used focus groups to identify user needs and preferences and to structure the material within a multimedia program. A prototype was taken forward in user tests followed by in-depth interviews. These led to the identification of problems such as logging into and out of the program and accessing the exercises. Participants enjoyed the computer training sessions and gained considerable satisfaction from learning a new skill that they previously thought was not feasible. Lopes et al [22] used interviews to analyze user needs and identify commonly misplaced items, such as keys, glasses, cell phones, and identity papers. Focus groups and user tests were then used to try out existing item locators and define the following system requirements of a new item locator prototype: ease of use, capacity for customization, low price, nonstigmatizing design, and being *fun* to use. The next step included user tests with the first prototype in which participants commented that they would prefer to be guided by a customized sound of a voice system to find an item.

#### Design of Existing or Future Technology

In 2 studies, feedback was gathered solely on the design of future technologies using qualitative methods only. In a study by Meiland et al [24], nonfunctional mockups were reviewed after discussing the potential functionalities of an integrated, assistive system in focus groups and interviews. Participants valued help in case of emergencies, navigation support, and the calendar function the most. The least preferred functionalities were activity support and picture phone dialing. McCabe and Innes [23] found that people with dementia and carers gave specific feedback on the form and features of a potential GPS design during focus groups (eg, waterproof watch style design with a range of colors). However, participants would have preferred to comment on an actual and active device rather than talking hypothetically, as it did not provide them with enough context.

In 3 studies, participants gave feedback on the design of an existing and functional technology. Freeman et al [17] analyzed observational data of people with dementia using 2 websites. These data helped to uncover 3 major problems: scrolling, nonrecognition of more information on a page, and getting stuck. There was a high degree of overall satisfaction with both sites measured through questionnaires. Kerkhof et al [26] interviewed residents after bench testing a memory aid (planning board). The majority of residents appreciated the use and function of the aid, but successful implementation was difficult because of installation errors, limited user friendliness, and lack of knowledge regarding the function and the use of the aid. Areas of focus for improvement include software program adaptation, additional technological applications, internet connectivity, accessibility, and addition of media. Finally, Klein et al [20] also analyzed observational data of the participants while testing 2 prototype devices. On the basis of the findings from these tests, a third prototype device was developed. Special attention was given to more personally relevant and engaging content, contextual factors, higher levels of immersions, and more control for the user.

### Feasibility and Piloting Phase (n=7)

A total of 7 studies included only the feasibility and piloting phase, which can be part of the design phase of the CeHRes roadmap [13-16,28-30]. In this phase, people with dementia were given the opportunity to try out a piece of technology in a pilot study or through field testing. Often, the aim was to gain insights into the usefulness of a device along with its acceptability and usability (Figure 2). In the majority of the studies (n=5), a mixed methods approach was adopted where participants were observed while using the device and feedback was obtained through semistructured interviews and questionnaires. Textbox 1 gives an overview of all the methods used in the feasibility and piloting phase.

Begum et al [16] used observations to investigate adherence to prompts from a robot, engagement with the robot, and how often a task was completed. Interviews and questionnaires provided information on the acceptance, ease of use, usefulness, and physical attributes of the assistive robot. Meiland et al [29] field tested an integrated digital prosthetic with multiple functionalities. Data on its usability were collected through behavioral observations, interviews, and questionnaires, and it was deemed to be user-friendly and useful, but there was a desire for more personalization and configuration of reminders.

Moyle et al [15] explored the acceptability of a telepresence robot using observations through video recordings and follow-up interviews. Participants indicated a positive social presence, which was also observed through the display of positive emotions. A similar methodology was adopted in another study by Moyle and Jones [14]. Observations through video recordings were used to describe the effectiveness of a virtual reality forest (VRF) on engagement, apathy, and mood states. Overall, the VRF was perceived to have a positive effect, but there were higher levels of fear and anxiety. Follow-up interviews were used to explore the experiences of using the VRF. Most participants reported positive perceptions and suggested making the experience more active.

Topo et al [30] used questionnaires to collect information on the functional ability of people with dementia. Through interviews, data were collected on the usage and usefulness of an existing music tool 2 weeks after installation in a care home. Most participants benefited from its use and had positive experiences. Some problems were reported with the sensitivity of the touch screen and the font size being limited because of the screen size.

Jamin et al [28] used a qualitative approach where participants were involved in usability testing and were observed while interacting with *VENSTER*. The content of VENSTER, which needs to provide enough context to be meaningful, was interesting and suitable for the participants. The study by Khosla et al [13] was the only study using a quantitative methodology in which participants were observed while interacting with a social robot to gain insights into emotional, visual, and behavioral engagement. In addition, user surveys were used to assess acceptability. The participants generally had a positive attitude toward social robots. Most of the participants gave high ratings in terms of the perceived usefulness and enjoyment of their experience with the robot.

### Development and Feasibility and Piloting Phase (n=3)

A total of 3 studies elaborated on both the development, and feasibility and piloting phase [18,25,27]. These studies systematically described the involvement of people with dementia over the course of each phase: the identification of user needs and wishes, determination of the design, and testing of a prototype version through a pilot test or field test. For each of these activities, a wide array of methods was applied, such as focus groups and interviews, workshops, and usability tests.

In the study by Span et al [18], the development phase consisted of interviews to identify needs and preferences for an interactive web tool and focus groups to discuss the results of the interviews and to make any additions to the problems and experiences shared. Several user requirements were identified, such as social contacts, daily activities, care, autonomy, involvement, and communication, specifically for the decision-making process. Paper mockups were discussed in focus groups to design the interactive prototype. Thereafter, individual user tests were organized to gather feedback on an interactive prototype regarding design, content, and user friendliness. Some participants found it difficult to comment on paper mockups but overall mentioned that information per screen and the number of screens should be decreased and the accuracy of language was of importance. For the feasibility and piloting phase, an interactive prototype was field tested to gain feedback on the user friendliness of the tool, the contentment of the participants, and how they valued the tool for decision making.

Martin et al [27] used interviews in the development phase to identify the main issues, risks and care needs arising during nighttime. The main themes included promoting independence, maintaining dignity, maximizing social inclusion, managing risk, and providing stimulation. In the feasibility and piloting phase, participants were involved in any of the 3 phases of iterative validation and evaluation of a prototype through technical system usage and interviews. The phases included testing for stability, usability, and integration within a full telecare system and the implementation of music and light. Participants liked the mobile component of the nighttime system and the easy navigation.

Davies et al [25] used both interviews and workshops to identify user needs in specific areas of cognitive reinforcement in the development phase. The following areas were identified by the participants: remembering, maintaining social contact, performing daily life activities, and enhanced feelings of safety. Interviews accompanied field testing in the feasibility and piloting phase. After trying out 4 prototypes, participants highlighted the need for personalization, less complex functionality, and extended use within the home environment.

### Evaluation Phase (n=1)

One study involved people through evaluation in a controlled trial [12]. Participants used an assistive system and filled in posttest questionnaires to assess impact. Despite no significant effects on impact, posttrial interviews and focus groups were used to assess qualitative impact, and participants found the system to be very useful but not user-friendly because of technical difficulties, including the unresponsiveness of touch screens and issues with gaining access. For people who had not used a touch screen before, the system was deemed unintuitive.

### Involving People With Dementia

#### Impact on the Developed Technology

In all but 5 studies [13,15,19,20,25], researchers directly reflected on the involvement of people with dementia in the development of the technology-based intervention. Researchers concluded that it was both necessary and feasible to involve people with dementia throughout the development process. In addition, Kerkhof et al [26] argued that it is not sufficient to respond to the needs of people with dementia by solely involving carers or staff members. This is further supported by Meiland et al [24] and Lopes et al [22] who found that exploring the user perspectives from various stakeholders, including people with dementia, is necessary to understand the problem and come up with possible solutions. Jamin et al [28] also emphasized that codesign with all stakeholders can make the overall experience more pleasurable but also more meaningful, as it allows the users to be kept at the center of the decision-making process and adaptations can be made to new insights as they emerge. In several studies, it was recognized that people with dementia continue to be one of the most excluded groups from research and the design of new services [6,21]. Possible reasons for this could be difficulties in recruitment or the cognitive impairment of people with dementia [16,29]. However, despite these challenges, all studies recommended the involvement of people with dementia in future studies, as this could lead to obtaining views on new concepts or ideas for technology and to more concrete feedback on the usability and user friendliness of a device. For instance, one study determined how to maximize website suitability for people with dementia after receiving feedback [17]. Another study adapted the appearance of a robot and made it more socially interactive [16]. Finally, people with dementia suggested that the interaction between end users and a virtual reality system could be improved by incorporating reminiscence within the tool [14].

#### Impact on the Person With Dementia

The positive effects of involvement on people with dementia themselves included the empowering effects of involvement that were evident in increased feelings of well-being, being able to voice opinions, learning a new skill through the use of technology, and an enhanced sense of control experienced by the majority of the participants [6]. Participants were also motivated to contribute to research and a better quality of life for future people with dementia [18,26]. No distress or adverse events from involving people with dementia were reported in any of the studies.

#### Outcomes of the PPI Consultations

PPI group members reflected on how to optimize involvement both in research and in the development of technology-based interventions and endorsed the guidelines (Multimedia Appendix 3) [6,12,18,21,23,26-28,30]. Additions were made to some guidelines, for example, there was consensus among members that researchers need to focus on individual research participants, which includes awareness of their type of dementia, any other relevant conditions, and any specialized knowledge of participants, which could further support the development of technology. Awareness among participants in terms of the relevance and positive effects of involvement for them was also important.

A friendly research environment was helpful to make people feel comfortable to ask questions in case they did not understand something. This is especially helpful when developing new technology, which can include some unknown aspects, and so researchers should also aim to avoid abbreviations and acronyms to avoid technology-related jargon. In addition, PPI group members suggested that researchers should present their materials at a PPI meeting before an actual research activity takes place to ensure the use of jargon is limited.

Involving people with dementia as early as possible in the development process and in multiple phases of development should lead to increased familiarity and a better understanding of the technology. Members were also positive about encouraging technology developers to interact directly with people with dementia but highlighted that a mediator (eg, a researcher) would be necessary to ensure a good level of understanding between people with dementia and the developers. A person with dementia also mentioned taking a technology into the community (eg, a memory café) to gather feedback, as this would allow for the technology to be used in a real-life setting.

Finally, a *Wizard of Oz* method was suggested by a researcher where participants interact with a working prototype but under the guidance of an unseen researcher. The 2 PPI group members with dementia mentioned that they would not have an issue with this in terms of ethics, and it was regarded as a good idea. This method could be used to limit the amount of errors.

## Discussion

The *Discussion* section comprises the third element of narrative synthesis: exploring relationships within and between studies.

### Summary and Interpretation of Findings

People with dementia can contribute effectively to the development of technology but are often excluded from research in this area. With the rise of innovative technology, there is a need for an overview of the current evidence regarding the involvement of people with dementia and recommendations on how to optimize this involvement in the development process. This is to ensure that the developed technologies are suitable and tailored toward the needs of the end users. This is the first narrative synthesis review to synthesize the findings from high-quality studies of involvement of people with dementia in developing technology-based interventions and has created best practice guidelines based on the evidence summarized below.

One of the strengths of this review is the strict inclusion criteria leading to the synthesis of high-quality papers. This further supports the robustness of the findings and the developed guidelines. Furthermore, the application of narrative synthesis in this systematic review allowed for a highly systematic approach to search for and make sense of the evidence. The underpinning theory, as part of the first element of narrative synthesis, helped define the research questions and the studies to be included in this review. In addition, the preliminary synthesis supported the tabulation of the findings, which is highlighted in the text, tables, and figures. This approach also proved helpful in converting the evidence into best practice guidelines by looking for relationships within and between the studies. Good examples of involvement were extracted and incorporated into the guidelines, which were modified by the input of the PPI group. This enabled the invaluable perspective of people with lived experience on the findings and helped strengthen the robustness of the synthesis and relevance of the guidelines.

The findings suggest that the involvement of people with dementia varies depending on the development stage and methods used, which is in line with previous research [4]. A large part of involving people with dementia revolves around identifying user needs and preferences. The majority of the studies included this aspect in their research and primarily used qualitative methods such as focus groups and interviews. The identification of needs often helped to prioritize the most pressing issues for people with dementia.

Another component is gathering feedback on either the prospective or existing design of a device. These activities mostly include qualitative methods while using observations and questionnaires. People with dementia play an active role in voicing their opinions and trying out the available prototypes. Once a piece of technology has been developed into a more refined version, the involvement of people with dementia shifts more toward the participants becoming the objects of study. In several studies, people with dementia were asked to use a piece of technology more rigorously during a field-testing phase accompanied by observations and to give feedback after the test phase. Interestingly, no studies involved the participation of people with dementia in the implementation phase.

These findings are congruent with findings from a review by Span et al [4]. However, in this review, we found studies that described more elaborately the involvement of people with dementia and demonstrated that it is feasible to include them throughout the entire development process rather than in a single phase. The involvement of people with dementia started with exploring their needs and gaining an understanding of the current problem, which led to the development and testing of various prototypes together with people with dementia to tailor it to their needs. These studies set a good example for future studies by applying various methods and obtaining in-depth data from people with dementia. The impact of the involvement is also evident as studies have provided examples of concrete pieces of feedback from people with dementia, which improved the developed technology. However, there is also an impact of involvement on the person himself or herself, as some studies have shown that the involvement of people with dementia can be empowering and lead to increased feelings of well-being [6]. Participants expressed the importance of being able to contribute to the research by voicing their own opinions [4,6,26]. None of the studies noted any distress caused by the involvement of people with dementia. This is helpful for future studies, as anticipated distress from trying out underdeveloped technology was seen as a reason to not include people with dementia in development [19].

Some challenges were described in the involvement of people with dementia, such as the risk of obtaining socially desirable answers [21,29]. However, this risk is not specific to this population and, in general, is not uncommon in research. Another challenge was obtaining in-depth feedback from participants, as the use of unfamiliar terms related to technology made it difficult for participants to comprehend the questions [30]. Jamin et al [28] emphasized the need for the involvement of multiple stakeholders but acknowledged that this adds a level of complexity to the design process, as researchers or developers would have to navigate various differing opinions. Despite these challenges, all studies recommended that people with dementia should be involved in developing technology and also to keep including relevant stakeholders such as (in)formal carers and technology developers where possible.

### Best Practice Guidelines (Narrative Synthesis Element 4: Assessing the Robustness of the Synthesis)

On the basis of the findings from the studies included in this review and the contributions from the PPI consultation meetings, best practice guidelines for the involvement of people with dementia in developing technology-based interventions were developed (Multimedia Appendix 3). A previous best practice model included in a systematic review by Di Lorito et al [35] served as an example to better organize the findings according to the goals of involvement, preparations, and the contributions from the PPI consultation meetings. A score can be allocated to each guideline depending on whether it has been fully met (score=2), partly met (score=1), or not met (score=0). The availability of 12 guidelines means that a total score of 24 can be achieved, indicating that each guideline has been met in full when developing a technology-based intervention for people with dementia.

Having the right prerequisites in place before involvement can help overcome the challenges and optimize the involvement of people with dementia. When it comes to the participants, prioritizing their well-being and ensuring that they are aware of the purpose and relevance of their involvement can help contribute to an enjoyable research experience [6,21]. Both the findings from this review and the suggestions from the PPI group members emphasized the need for skilled researchers and the need for a comfortable research environment. Researchers need to take time to get to know participants, and PPI group members added that researchers should be aware of any specialized knowledge of people with dementia before their involvement. This could strengthen their contributions, and it would easily enable them to become coresearchers. Furthermore, determining the goal of involvement and where it is best suited in the development process will help avoid wasting time of people with dementia [27].

Keeping in line with this, multiple methods for involvement need to be considered to obtain the most optimal feedback, and where possible, multiple phases of development should be included. This was confirmed by the PPI group members, and in addition to this, early involvement of people with dementia was considered helpful, as it would also help to identify their own needs and ideas for technology. The latter is crucial in some of the studies included in this review, in which people with dementia are involved in needs assessments and prioritizing areas for functional improvement before moving on to prototype development. It is also recommended to involve all relevant stakeholders and allow interaction between them to obtain a well-rounded view from several user perspectives but also to enable people with dementia to become part of the research and development team [26,28].

During the involvement of people with dementia, the research experience can be further enhanced if participants are able to learn a new skill involving technology [6,18]. This can lead to increased motivation and feelings of empowerment. In addition, the use of appropriate terminology can be helpful in obtaining meaningful and more in-depth answers [30]. Technology must meet an acceptable standard of stability and reliability when evaluating its impact [12]. This can help to avoid frustration among participants and to avoid missing out on essential feedback. PPI group members agreed that it would be more useful to use functional devices during testing and added that the technology should be compatible with different platforms if applicable (eg, a computer or a mobile phone). However, members also reflected on the *Wizard of Oz* method and the idea of an unseen researcher operating the device from a distance while people with dementia would interact with it. This method could potentially function as a good alternative where paper-based prototypes are not suitable and fully functional prototypes are not available. After involvement has taken place, it is advisable to keep participants up to date regarding further development or implementation of the new technology.

**Figure 3:**
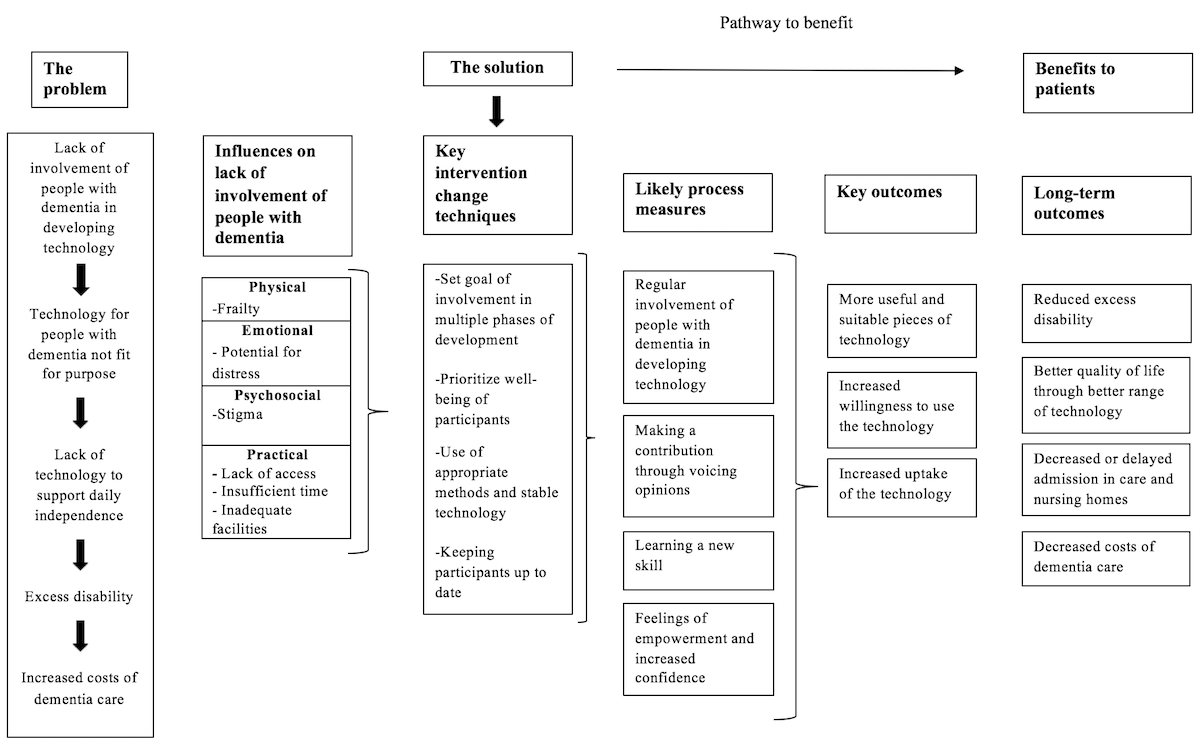
Optimizing the involvement of people with dementia in developing technology-based interventions: logic model.

Figure 3 includes a logic model based on the findings from this review and the best practice guidelines. It describes the current problem of a lack of involvement of people with dementia in developing technology and how this can be remedied through key intervention change techniques, such as setting goals of involvement and using appropriate methods. This will lead to important short- and long-term outcomes, including more useful pieces of technology and decreased costs of dementia care.

### Limitations (Narrative Synthesis Element 4: Assessing the Robustness of the Synthesis)

This review included very few studies that involved people with dementia in multiple stages of technology development. Furthermore, although this review did not focus on the passive involvement of people with dementia (eg, in large-scale RCTs), few studies allowed for impact evaluation and subsequent sharing of feedback, such as in the study by Hattink et al [12]. Finally, no studies were found that included the involvement of people with dementia in the implementation phase of development.

The definition of involvement in a development process was partly based on previous research and therefore only included studies in which people with dementia played an active part in development or were able to give feedback. This might have caused the exclusion of other potentially relevant studies, which involved people with dementia through other methods, which is a limitation of this review. Another limitation is the focus on English language peer-reviewed journal papers only, which may have led to the exclusion of other potentially relevant content.

### Future Research

To develop more tailored technology and explore the possible roles for people with dementia in other phases, future studies should expand on the level of involvement of people with dementia. People with dementia should be coresearchers or advisors and be made an integral part of the research team and the study. This would enable the same group of people with dementia to consistently provide feedback from the early stages of development (eg, formulating the problem) toward the mid stages and end stages (eg, design and implementation). Considering the lack of studies focusing on the implementation phase, future research should explore the role of people with dementia in both the implementation and dissemination of a new technology. In addition, in the majority of the studies, the researcher often acts as a mediator between the person with dementia and the technology developer. However, future studies could aim to facilitate direct knowledge transfer between the two for the technology developers to receive raw feedback.

### Conclusions

Over time, studies have involved people with dementia more rigorously in developing technology; however, technologies still need to be more tailored to the needs and preferences of people with dementia. To do this, people with dementia need to be given an active role in the development of technology, so they can have the opportunity to voice their thoughts and opinions. This narrative synthesis review has shown that it is feasible for people with dementia to assume a more active role throughout the development process from discussing and commenting to tryouts and testing. The involvement of people with dementia is associated with several benefits, namely, the development of better and more useful technology, an improved uptake of the technology, and an increased willingness to use the technology. In addition, the evidence-based best practice guidelines were deemed to be relevant by PPI group members and will help support future researchers, technology developers, and people with dementia to optimize involvement when developing technology (Textbox 2). This will not only ensure that future technology-based interventions are suitable but will also allow people with dementia to feel empowered by making an effective contribution to technology development and research in general.

**Summary of best practice guidance for involving people with dementia in developing technology.**

Prepare for involvement:

- Make this a positive experience for participants by creating a friendly environment, where people can ask questions and feel supported
- Involve a variety of stakeholders and users to collect a range of feedback and perspectives
- Ensure all practicalities for involvement are in place to meet the needs of participants
- Participants should be made aware of the purpose and relevance of their involvement to meet their expectations and encourage honest feedback
- Explore the available methods for collecting feedback and select the ones best suited for the goal of involvement

Practice involvement:

- Use appropriate terminology and words when asking questions to promote understanding and generate more in-depth feedback
- Offer participants the opportunity to learn a new skill through their involvement to enhance well-being and empowerment
- Involve participants throughout the development process to create a more suitable piece of technology for wider uptake
- Keep participants informed after their involvement so they can stay up to date on further development and implementation of the technology

## Appendix

Multimedia Appendix 1 Main characteristics of the included studies.

Multimedia Appendix 2 Methodological quality of the included qualitative studies.

Multimedia Appendix 3 Best practice guidelines for the involvement of people with dementia in developing technology-based interventions.

## Abbreviations

CASP: Critical Appraisal Skills Programme
CeHRes: Centre for eHealth Research
CINAHL: Cumulated Index to Nursing and Allied Health Literature
EMBASE: Excerpta Medica database
MEDLINE: Medical Literature Analysis and Retrieval System Online
MRC: Medical Research Council
PPI: patient and public involvement
PROSPERO: International Prospective Register of Systematic Reviews
RCT: randomized controlled trial
VRF: virtual reality forest
