# Supplementary material for: Involvement of People With Dementia in the Development of Technology-Based Interventions: Narrative Synthesis Review and Best Practice Guidelines
Source: J Med Internet Res. 2020 Dec 3;22(12):e17531. doi: 10.2196/17531 (PMC7746489; doi:10.2196/17531)
Supplement: Multimedia Appendix 1 [file jmir_v22i12e17531_app1.docx]

|  | | | | | | |
| --- | --- | --- | --- | --- | --- | --- |
| **Author & year** | **Country** | **Study sample** | **Study design** | **Description technology–based intervention** | **MRC phase of development (CeHRes phase)** | **Number/percentage of quality criteria fulfilled** |
| **1. Begum et al [16]** | Canada | PwD (n = 5),  carers (n = 5). | Mixed methods | Assistive mobile robot  Aim: to help PwD with activities of daily living such as hand washing and tea making. | Feasibility/piloting (design) | 7 |
| **2. Boman et al [21]** | Sweden | PwD (n = 6),  carers (n = 10),  occupational therapists (n = 8). | Qualitative | Easy to use videophone for PwD  Aim: to support PwD to make videophone calls without assistance. | Development (value specification) | 9 |
| **3. Davies et al [25]** | Sweden, Netherlands, United Kingdom | PwD (n = 17),  carers (n = 17). | Qualitative | Touch screen devices  Aim: to support independent living in four domains:  1. Remembering 2. Maintaining social contact 3. Performing daily life activities 4. Enhanced feelings of safety. | Development and feasibility/piloting (contextual inquiry, value specification, design) | 7 |
| **4. Freeman et al [17]** | United Kingdom | PwD (n = 5). | Mixed methods | Two prototype informational websites for PwD  Aim: to produce recommendations for the next stage of the design process. | Development (value specification, design) | 9 |
| **5. Hanson et al [6]** | Sweden | Development group:  PwD (n = 7), carer (n = 1).  Test group:  PwD (n = 19), relatives (n = 12). | Qualitative | The ACTION “Living with Dementia” multimedia education and support program consisting of the Life Story Book, the Diary, and the Family Tree  Aim: to provide PwD and family carers living at home with early information, education, and support. | Development (contextual inquiry, value specification, design) | 9 |
| **6. Hattink et al [12]** | Belgium, Germany, the Netherlands | PwD/MCI (n = 42),  informal carers (n = 32),  professional home-care workers (n = 6). | Mixed methods | A fully integrated, multifunctional system consisting of three subsystems (Rosetta)  Aim: to help people with MCI and dementia in performing the daily activities they indicated to be of importance (eg, reminders for activities, support in recreational activities and social contact, autonomous surveillance). | Evaluation (summative evaluation) | 7 |
| **7. Jamin et al [28]** | The Netherlands | PwD (n = 10), informal carers (n = 2),  activity supervisors (n = 2),  manager (n = 1), client representatives (n = 5). | Qualitative | An interactive artwork for nursing home residents (VENSTER)  Aim: to help nursing home residents to connect with the outside world through an interactive physical window and therefore to help decrease feelings of isolation. | Feasibility/piloting (design) | 7 |
| **8. Kerkhof et al [26]** | The Netherlands | PwD (n = 6)  informal carers  (n = 5),  staff members  (n = 6). | Qualitative | Memory aid consisting of digital planning boards  Aim: to structure and support the daily activities for PwD. | Development (contextual inquiry, value specification, design) | 9 |
| **9. Khosla et al [13]** | Australia | PwD (n = 115). | Quantitative | Social robot (Matilda)  Aim: to support emotional well-being through the delivery of diversion therapy services to older PwD. | Feasibility/piloting (design) | 65% (Downs & Black) |
| **10. Klein et al [20]** | Germany | Contextual inquiry: PwD (n = 50),  carers (n = 18), relatives (n = 17).  Test 1: PwD (n = 24),  Test 2: PwD (n = 25), Test 3: PwD (n = 4), cognitive impairment (n = 2). | Qualitative | Multimedia approaches such as haptic and virtual reality artefacts (the Jukebox, Pyramid, and the Binoculars).  Aim: to improve well-being through providing reminiscence therapy. | Development (contextual inquiry, value specification, design) | 7 |
| **11. Lopes et al [22]** | France | PwD/MCI  (n = 46),  informal carers  (n = 35), formal carers  (n = 29) across five studies. | Qualitative | Item locator device (TROUVE system)  Aim: to help older adults with MCI and AD to find misplaced or “lost” personal items at home. | Development (contextual inquiry, value specification, design) | 9 |
| **12. Martin et al [27]** | United Kingdom | PwD (n = 8),  healthy older people (n = 12). | Qualitative | NOCTURNAL system  Aim: to address disturbed sleep patterns and night time wandering of PwD in an assisted living home. | Development and feasibility/piloting (contextual inquiry, value specification, design) | 7 |
| **13. McCabe et al [23]** | United Kingdom | PwD (n = 12),  carers (n = 3),  healthy older people (n = 5). | Qualitative | A GPS/safe walking device  Aim: to give more confidence to go out independently and to help carers locate the person with dementia if needed. | Development (contextual inquiry, value specification) | 9 |
| **14. Meiland et al [29]** | The Netherlands, Sweden, United Kingdom | Field test 1: PwD (n = 16), carers (n =16)  Field test 2: PwD (n = 14), carers (n = 13)  Field test 3: PwD (n = 12), carers (n =12). | Mixed methods | COGKNOW Day Navigator  Aim: to give support for PwD in reminding, social contact, daily activities and feelings of safety. | Feasibility/piloting (design) | 8 |
| **15. Meiland et al [24]** | Germany, the Netherlands | PwD/MCI (n = 14),  informal carers (n = 13), formal carers (n = 6), dementia experts (n = 9), care partners (n = 7), volunteer (n = 1). | Qualitative | New version of the “Rosetta” system  Aim: to combine three systems - COGKNOW Day Navigator (CDN), the EMERGE system and the Unattended Autonomous Surveillance system (UAS) – for PwD and carers, providing support in daily functioning, monitoring patterns in daily behavior and to automatically detect emergency situations. | Development (contextual inquiry, value specification, design) | 9 |
| **16. Moyle et al [15]** | Australia | PwD (n = 5),  carers (n = 5),  health professionals (n = 12). | Mixed methods | Telepresence robot (Giraff)  Aim: to support the connection and engagement of PwD and family members though video calls. | Feasibility/piloting (design) | 9 |
| **17. Moyle et al [14]** | Australia | PwD (n = 10),  family members (n = 10),  care staff (n = 9). | Mixed methods | Virtual Reality Forest on large interactive screen  Aim: to improve quality of life through improving engagement, apathy, and mood states. | Feasibility/piloting (design) | 8 |
| **18. Orpwood et al [19]** | United Kingdom | PwD (n = 26, 16 living at home and 10 living in care homes). | Qualitative | Four items developed from a “wish list”:  1. Music player 2. Window on the world: to reduce social isolation 3. A conversation prompter  4. Sequence support for several tasks eg. sending a letter, finding a TV program.  Aim: to support quality of life of PwD. | Development (contextual inquiry, value specification, design) | 7 |
| **19. Robinson et al [7]** | United Kingdom | Scoping stage:  PwD (n = 10), carers (n = 11).  Participatory design stage: PwD and carers (n = 22).  Prototype development stage: PwD (n = 2), carer (n = 1). | Qualitative | Two prototype tracking devices: armband and electronic notepad  Aim: to facilitate independence for PwD and to facilitate mutual communication between PwD and their families. | Development (contextual inquiry, value specification, design) | 7 |
| **20. Span et al [18]** | The Netherlands | Interviews: PwD (n = 23)  Focus groups: PwD (n = 18)  Usability tests: PwD (n = 3)  Field study: PwD (n = 4). | Qualitative | The DecideGuide (a web tool)  Aim: to help PwD, informal caregivers, and case managers make shared decisions. | Development and feasibility/piloting (contextual inquiry, value specification, design) | 9 |
| **21. Topo et al [30]** | Finland,  Norway, Ireland,  UK | PwD (n = 23),  staff members  (n = 17). | Mixed methods | The Picture Gramophone (PG) multimedia program  Aim: to be used by PwD, to stimulate them and to give them pleasure. | Feasibility/piloting (design) | 7 |
| *PwD = people with dementia, MCI = mild cognitive impairment, AD = Alzheimer’s Disease* | | | | |  |  |
