# Supplementary material for: Involvement of People With Dementia in the Development of Technology-Based Interventions: Narrative Synthesis Review and Best Practice Guidelines
Source: J Med Internet Res. 2020 Dec 3;22(12):e17531. doi: 10.2196/17531 (PMC7746489; doi:10.2196/17531)
Supplement: Multimedia Appendix 2 [file jmir_v22i12e17531_app2.docx]

| CASP Qualitative Checklist (https://casp-uk.net/casp-tools-checklists/) | Begum et al [16] | Boman et al [21] | Davies et al [25] | Freeman et al [17] | Hanson et al [6] | Jamin et al [28] | Kerkhof et al [26] | Klein et al [20] | Lopes et al [22] | Martin et al [27] | McCabe et al [23] | Meiland et al [29] | Meiland et al [24] | Moyle et al [15] | Moyle et al [14] | Orpwood et al [19] | Robinson et al [7] | Span et al [18] | Topo et al [30] |
| --- | --- | --- | --- | --- | --- | --- | --- | --- | --- | --- | --- | --- | --- | --- | --- | --- | --- | --- | --- |
| 1. Clear statement of aims? | + | + | + | + | + | + | + | + | + | + | + | + | + | + | + | + | + | + | + |
| 2. Qualitative methodology appropriate? | + | + | + | + | + | + | + | + | + | + | + | + | + | + | + | + | + | + | + |
| 3. Research design appropriate? | + | + | + | + | + | + | + | + | + | + | + | + | + | + | + | + | + | + | + |
| 4. Recruitment strategy appropriate? | + | + | + | + | + | + | + | - | + | - | + | + | + | + | - | + | + | + | - |
| 5. Data collected in a way that addressed the research issue? | + | + | + | + | + | + | + | + | + | + | + | + | + | + | + | + | + | + | + |
| 6. Has the relationship between researcher and participants been adequately considered? | - | - | - | - | + | - | - | - | - | - | - | - | - | - | - | - | - | - | - |
| 7. Ethical issues taken into consideration? | - | + | - | + | + | - | + | + | + | + | + | + | + | + | + | - | - | + | - |
| 8. Data analysis sufficiently rigorous? | - | + | - | + | - | - | + | - | + | - | + | - | + | + | + | - | - | + | + |
| 9. Clear statement of findings? | + | + | + | + | + | + | + | + | + | + | + | + | + | + | + | + | + | + | + |
| 10. How valuable is the research? | + | + | + | + | + | + | + | + | + | + | + | + | + | + | + | + | + | + | + |
| Total | 7 | 9 | 7 | 9 | 9 | 7 | 9 | 7 | 9 | 7 | 9 | 8 | 9 | 9 | 8 | 7 | 7 | 9 | 7 |

+ = criterion met; - = criterion not met
