# Supplementary material for: Involvement of People With Dementia in the Development of Technology-Based Interventions: Narrative Synthesis Review and Best Practice Guidelines
Source: J Med Internet Res. 2020 Dec 3;22(12):e17531. doi: 10.2196/17531 (PMC7746489; doi:10.2196/17531)
Supplement: Multimedia Appendix 3 [file jmir_v22i12e17531_app3.docx]

| **Guideline** | **Goals to be achieved** | | **Preparation** | | **PPI additions** | |
| --- | --- | --- | --- | --- | --- | --- |
| **Prior to involvement** | | | | | | |
| **1.** The well-being of people with dementia should be prioritised [6]. | - Ensure a positive research experience for participants. | | - Involve skilled researchers/practitioners. - When working in groups, ensure these are small in size (max. eight people with dementia). - Allow for time to get to know the participants. | | - Researchers need to be aware of the type of dementia and any other relevant conditions that might influence participation. - The positive aspects of participating need to be highlighted. - A friendly, light hearted environment is needed so participants feel comfortable to ask questions about things they do not understand. | |
| **2.** People with dementia should be made aware of the purpose of their involvement [21]. | - Decrease the risk of socially desirable answers. | | - Explore participants’ expectations regarding their involvement. - Encourage participants to be honest and critical. - Give participants the opportunity to be co-researchers. | | - Researchers need to highlight the relevance of participation. | |
| **3.** Researchers need to set the goal of involvement and take stock of when and where in the development process its best suited [27]. | - Ensure the involvement is optimal and meaningful. | | - Determine which stage of development the technology is currently at. - Assess what kind of information is needed from participants. | | - Participants should be involved as early as possible. - Hone in on specialised knowledge of participants (eg. prior experience with technology development). | |
| **4.** Involve people with dementia in several phases such as the development, validation and evaluation of technology [18, 27]. | - Wider uptake of the technology, and continued use by target population. | | - Get familiar with the phases of technology development by applying existing frameworks. | | - Involve the same participants in multiple phases. | |
| **5.** Identify the best methods for providing feedback. A combination of several methods should be considered. | - Obtain diverse and in-depth data which is representative of the participants’ feedback. | | - Determine the type of data which needs to be obtained. - Qualitative methods such as focus groups and interviews are appropriate to identify needs and user requirements or to gain feedback after bench-testing a technology. - Quantitative methods such as surveys, user tests are appropriate when field-testing a technology. | | - Gather feedback in community settings such as memory cafes. | |
| **6.** Involve all relevant stakeholders including people with dementia, informal carers, staff members, technology developers and where possible allow interaction between stakeholders [26, 28]. | - Obtain a well rounded view through the feedback from several user perspectives. - Accentuate or confirm value of findings across the groups. - Enable people with dementia to be part of a bigger team. | | - Organise various research activities for each stakeholder group and explore where appropriate activities may be combined. - Create an opportunity for people with dementia to meet technology developers or other stakeholders. | | - A mediator can help to promote the same level of understanding between IT developers and service users. | |
|  |  | |  | |  | |
| **7.** Ensure all the practicalities for involving people with dementia are in place such as sufficient time and a suitable location [6, 18]. | - Build a trusting relationship between participants and research team. - Create a familiar and supportive environment. - Increase accessibility to the research activity. | | - Factor in time dedicated to socialising and getting to know each other. - Funders can facilitate researchers’ responsibility by counting in extra time for participation of people with dementia. - Location should include disabled access, car parking, public transport, and amenities such as a kitchen or pantry, and hot drinks. | | - A long session can lead to fatigue. Sprints of 45 minutes could be appropriate depending on people’s preferences. | |
| **During involvement** | | | | | | |
| **8.** Use appropriate terminology and words when asking questions [30]. | - Obtain more meaningful answers. - Avoid confusion. | | - Adopt multiple methods for data collection for example observations paired with interviews**.** - Develop clear and easy to answers questions. - Avoid the use of uncommon or unfamiliar technology jargon. - Check whether terms/questions are understood by all participants. | | - Present any materials to be used at a PPI meeting prior to the actual research activity. - Avoid the use of acronyms and abbreviations. | |
| **9.** When assessing the design or usability of a technology, gather feedback from people with dementia on working prototypes which can be operated by the users rather than paper prototypes [23]. | - Ease of providing feedback for people with dementia. - Create a better understanding of the technology to be developed. | | - Create digital mock-ups. - Have the device present and switched on at the time of asking questions so participants can test it. | | - Wizard of Oz method which includes an unseen researcher operating the device from a distance, could be an alternative. | |
| **10.** When evaluating the impact of a technology on daily life, ensure the system meets an acceptable standard of stability and reliability [12]. | - Avoid frustration among participants due to faulty technology. - Avoid missing out on essential feedback. | | - Have multiple rounds of development (sprints) to iron out some faults. - Bench-test the technology before it is used over a longer period of time. | | - If possible and applicable, offer the technology on different platforms (eg, computers, touch-screen devices). | |
| **11.** Offer people with dementia the opportunity to learn a new skill through their involvement [6, 18]. | - Contribute to the overall experience of involvement. - Empower and enhance well-being of people with dementia by enabling them to learn a skill which previously they may have thought was not feasible. | | - Allow time for people with dementia to get familiar with the technology. - Where possible, include a training session on how to use a technology (eg, a tablet). - Encourage people with dementia to learn about the technology either by themselves or together with others. | |  | |
| **After involvement** | | | | | | |
| **12.** Allow for people with dementia to receive updates on the development and implementation of the technology. | | - Inform participants how their involvement and feedback supported development. | | - Send out newsletters including updates and share contact details of research team. - Where possible, give access to the developed technology. - If necessary, make sure technology is kept up to date and involvement of people with dementia takes places accordingly. | |  |
